# Impairment of the activin A autocrine loop by lopinavir reduces self-renewal of distinct human adipose progenitors

**DOI:** 10.1038/s41598-017-02807-9

**Published:** 2017-06-07

**Authors:** Christophe Ravaud, Martin Paré, Stéphane Azoulay, Christian Dani, Annie Ladoux

**Affiliations:** 1 Université Côte d’Azur, CNRS, INSERM, iBV France; 2 Université Côte d’Azur, CNRS, ICN, France

## Abstract

Maintenance of the adipose tissue requires a proper balance between self-renewal and differentiation of adipose progenitors (AP). Any deregulation leads either to fat overexpansion and obesity or fat loss and consequent lipodystrophies. Depending on the fat pad location, APs and adipocytes are heterogeneous. However, information on the pharmacological sensitivity of distinct APs to drugs known to alter the function of adipose tissue, especially HIV protease inhibitors (PIs) is scant. Here we show that PIs decreased proliferation and clonal expansion of APs, modifying their self-renewal potential. Lopinavir was the most potent PI tested. Decrease in self-renewal was accompanied by a reduced expression of the immediate early response gene IER3, a gene associated with tissue expansion. It was more pronounced in chin-derived APs than in knee-derived APs. Furthermore, lopinavir lowered the activin A–induced ERK1/2 phosphorylation. Expressions of the transcription factor EGR1 and its targets, including *INHBA* were subsequently altered. Therefore, activin A secretion was reduced leading to a dramatic impairment of APs self-renewal sustained by the activin A autocrine loop. All together, these observations highlight the activin A autocrine loop as a crucial effector to maintain APs self-renewal. Targeting this pathway by HIV-PIs may participate in the induction of unwanted side effects.

## Introduction

The adipose tissue (AT) represents the most adaptable tissue of an organism. It exists as functionally different depots that display opposite functions to fulfill the energy demand. In response to elevated calorie intake, white adipose tissue expansion allows energy storage as triglycerides. It represents the most abundant adipose tissue in adult humans. In contrast, brown adipose tissue is a key thermogenic organ able to produce heat from nutriments by uncoupling respiration from ATP synthesis. It surrounds the deepest organs^1^ and represents the lesser part of adipose tissue. White AT is present all over the human body and is composed of distinct depots that are heterogeneous in terms of cellular composition, proliferation and differentiation^2, 3^.

The adipose progenitor (AP) pool hosted within the adipose tissues is crucial for AT development and to form new fat cells upon appropriated stimulus that induce adipocyte differentiation. This process is essential because like most mature and specialized healthy cells, adipocytes are generated through differentiation of progenitor cells as they do not divide *in vivo*. Thus, the AP pool is directly correlated with adipose tissue development and linked to fat mass^4^. Our lab has recently shown that the AP pools are derived from distinct embryonic origins. In rodents, while the intra-abdominal depots originate from mesoderm, those from the face display a neuro-ectodermal origin^5^. This feature is also encountered in humans since APs from chin and knee do not express the same homeobox genes (*HOX*) code. Indeed, they produce adipocytes displaying different phenotypes: upon adipose differentiation, chin APs give rise preferentially to cells with brown/brite adipocytes features while knee APs are prone to generate white adipocytes^6^. All these observations suggest that the different fat depots are not equal either in terms of structures but also for physiological and metabolic functions. As AP cells need to perpetuate all life long, this pool is located in niches that maintain their intrinsic properties and control the self-renewal throughout adult life. Many factors present in the adipose tissue microenvironment are involved in a proper regulation of the balance between self-renewal and differentiation of these cells, including activin A which maintains the AP pool through autocrine and paracrine mechanisms^7, 8^. In addition, we recently identified the immediate early response 3 (*IER3*)^9^ as an important gene to control the activin A-induced expansion of this pool of cells^7^. *IER3* is induced in response to distinct microenvironmental effectors that are susceptible to be modulated by therapeutic treatments. However, information linking the sensitivity of the distinct AP pools to drugs that may affect fat depot development is limited. Individual responses of APs to distinct medicines are not well defined so far.

Treatment of AIDS patients with antiretroviral therapy (ART) dramatically improved the life of patients, their immune functions and has reduced morbidity and mortality resulting from AIDS-related complications. Several classes of antiretroviral drugs are used to treat HIV-infected patients. Among them, proteases inhibitors (PIs) prevent the HIV protease to cleave precursor proteins that are essential to form infectious viral particles. Unfortunately, this therapeutic class of molecules displays unwanted side effects which are prejudicial for adhesion of patients to the treatment. In various regimens, PIs have been associated with abnormal fat distribution and selective loss of fat depots, dyslipidemia, hypertriglyceridemia, insulin resistance and an increased risk of cardiovascular diseases^10, 11^. ART therapy has been responsible for the development of acquired lipodystrophies that represents the most predominant type in the population^12^ as compared to genetically acquired disorders^13^. Despite the development of new and safer molecules^14^, these effects prevail as 57% of the 2–18 years-old HIV-positive population treated with ART displays lipodystrophy^15^.

ART therapy induces a loss of the subcutaneous fat, notably within the depots of the face, and an excess deposition in the neck and the abdomen, indicating that all the fat depots are not affected in a similar way^16^ and these differences in sensitivity were reported within the same person. The heterogeneity in these various responses may result from intrinsic differences within the precursor cells.

Several reports point out that PIs impair adipocyte differentiation reducing then the number of fat cells generated from APs^17^. Of note, the fat loss in AIDS patients worsens with ongoing ART therapy and discontinuation of the treatment neither inverted this situation nor its associated complications. This observation implies that not only the differentiation process is altered by ART therapy. Fewer reports describe the effects of PIs on AP cells issued from distinct fat depots and information on the process leading to a modification of the intrinsic properties of the AP pool in response to ART therapy is rather scant.

A better comprehension of the molecular alterations induced by HIV-ART molecules on APs represents a valuable approach to illustrate the specificity of the distinct depots and to identify the signaling pathways important for adipose tissue development. It also allows a better comprehension of PIs-induced lipodystrophy development that may be of interest to improve the panel of therapeutic options.

In this study, we used HIV-PIs to better understand how self-renewal and differentiation were regulated in APs of different origins. We show that, in addition to our reference model (hMADS cells^18^), AP cells isolated from distinct fat depots display heterogeneous sensitivities to HIV-PIs. Lopinavir (LPV) is a peptide mimetic HIV-protease inhibitor. It altered proliferation of APs, chin-derived APs being the most sensitive, and it diminished differentiation of some but not all APs. In contrast, darunavir (DRV), a nonpeptidic antiretroviral protease inhibitor very efficient to decrease the activity of the HIV protease, reduced APs differentiation at high doses and displayed more moderate effects on proliferation. The expression of *IER3* was altered by LPV treatment. In addition, the activinA-induced ERK 1/2 phosphorylation was blunted thus promoting an alteration of the downstream signaling pathway. It especially lowered the expression of the early growth response 1 gene *EGR1*, a transcription factor also known as NGFI-A, Krox-24, ZIF268 and TIS8 which is highly dependent on ERK phosphorylation in self-renewing stem cells^19, 20^. *EGR1* controls important cellular processes including survival, cell growth^21, 22^ and appears as a determinant gene targeted by LPV. This cascade of events decreased activin A production and secretion impairing then its ability to maintain self-renewal, either in a paracrine or in an autocrine manner.

All together our results show that alterations of self-renewal properties occurred predominantly in the AP pool issued from the adipose depots of the face in response to LPV and they point out that LPV targets the activin A autocrine loop which is crucial to maintain the AP pool.

## Results

### Differentiation of APs derived from different adipose depots in presence of PIs is not equivalent

#### Lopinavir but not darunavir, altered adipose progenitors differentiation

**Figure 1:**
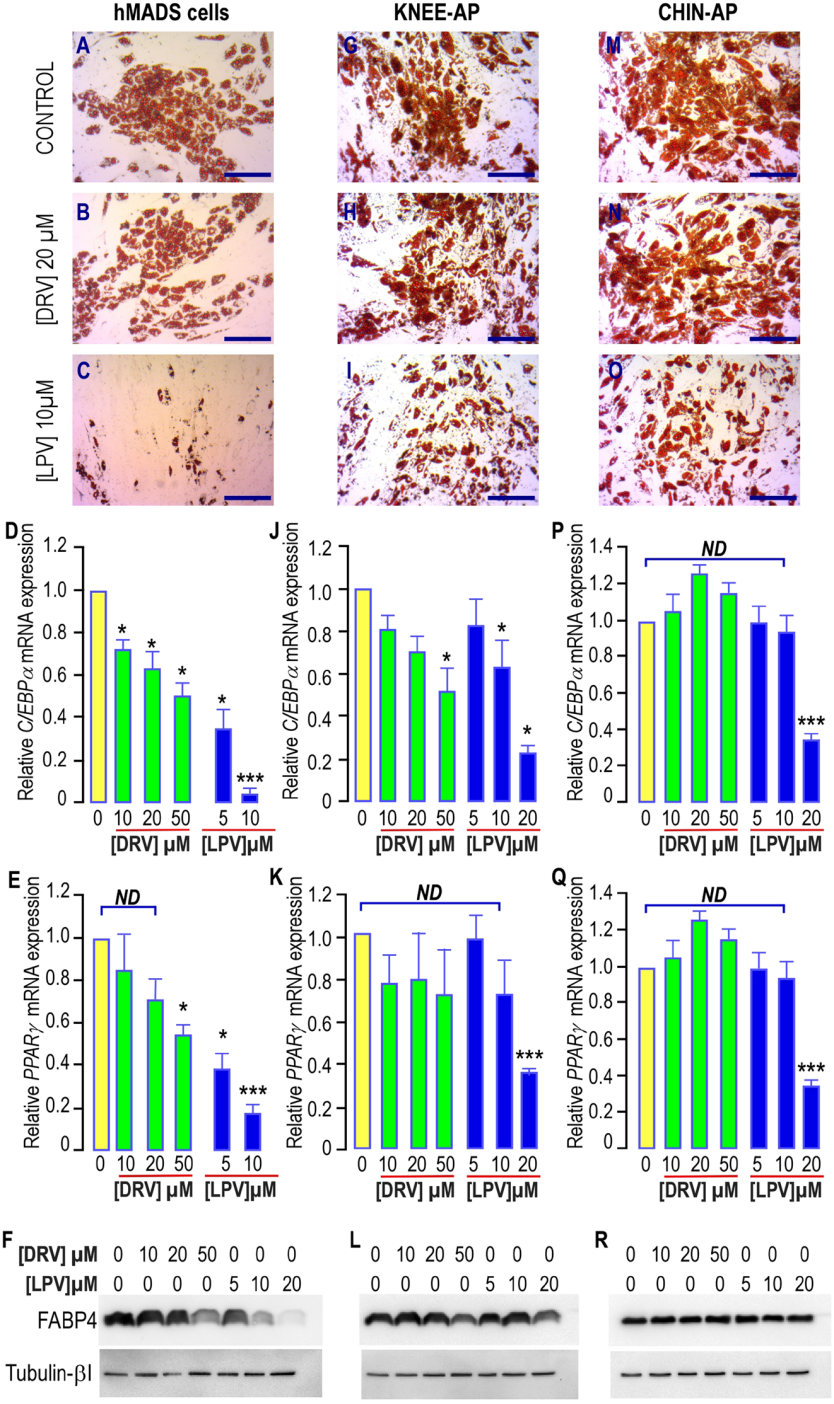
Effects of PIs treatment on differentiation of hMADS cells, chin and Knee APs. (**A**–**C**,**G**–**I**,**M**–**O**) Adipocyte differentiation in hMADS, knee or chin APs. After induction of differentiation for 17 days in absence or presence of PIs at the indicated concentrations, cells were fixed and further stained with Oil Red O. Differentiated cells displayed red staining within the lipid droplets. (**D**–**E**,**J**–**K**,**P**–**Q**) Expression of the adipogenic markers *C/EBPα* and *PPARγ* after 17 days of differentiation in absence or presence of increasing concentrations of PIs. Marker expression was assessed by real-time RT-PCR and normalized for the expression of *36B4* mRNA. Marker expression was measured in cells grown in the differentiation medium in absence of PIs (yellow bars) or in presence of DRV (green bars) or LPV (blue bars). The means ± SEM. were calculated from 3 (hMADS and knee) and 5 (chin) independent experiments, with determinations performed in duplicate (*p < 0.05, **p < 0.01, ***p < 0.001). (**F**,**L**,**R**) Expression of the adipogenic marker FABP4 after 17 days of differentiation in absence or presence of increasing concentrations of PIs. FABP4 expression was measured in cells grown in the differentiation medium in absence of PIs or in presence of increasing concentrations of DRV or LPV. Expression of Tubulin-βI used as a loading control (lower panel) and of FABP4 (upper panel) was analyzed by Western blot using specific antibodies. A typical autoradiograph is shown.

We measured the impact of DRV treatment on hMADS cells differentiation and compared it to LPV which is known to affect negatively the adipocyte maturation. We used concentrations in the range of those measured in the plasma of PI-treated patients. The therapeutic range for DRV was 10 µM onwards^23^ and it was comprised between 2–12 mg/ml, i.e. 3.2–19 µM, for LPV^24^. Indeed treatment of hMADS cells incubated with the differentiation cocktail in presence of 20 µM DRV allowed the apparition of lipid droplets as shown by Oil Red O staining (Fig. 1A and B) indicating that DRV is a weak inhibitor of adipose differentiation. In contrast differentiation was dramatically affected by a 10 µM LPV-treatment (Fig. 1C), indicating that the cells responded differently to these two drugs. Larger magnifications are presented in supplementary Fig. 1. These results were further confirmed by analysis of adipose differentiation markers. After 17 days of differentiation in hMADS cells, we observed that DRV significantly decreased the expression of genes important for adipocyte differentiation in a dose dependent manner, such as the transcription factors *C/EBPα* and *PPARγ* (Fig. 1D–E).In addition, LPV reduced the protein expression of the adipose marker FABP4 (Fig. 1F). Note that LPV treatment led to stronger effect while using lower concentrations as compared to DRV.

#### APs from distinct fat depots displayed different sensitivity to LPV’s effect

We determined the response of APs isolated from two distinct adipose depots from the same donor (chin and knee)^6^ to the inhibitory effect of PIs on the differentiation process.

We noted that adipose differentiation occurred in all APs cells treated or not with 20 µM DRV as shown by oil Red O staining (Fig. 1G,H,M,N) while LPV impacted this mechanism in a larger extend without abolishing it (Fig. 1I and O). In good correlation with our observations on hMADS cells, marker analysis revealed a dose-dependent impairment of differentiation when it was carried out in presence of DRV at concentrations higher than 20 µM in APs from knee (Fig. 1J). This result was further confirmed by protein analysis (Fig. 1l). In contrast, no effect was observed on APs derived from chin even at 50 µM DRV and at 10 µM LPV, a concentration that was detrimental for hMADS cells and knee-APs differentiation (Fig. 1D,P and Q). Here again, LPV did not prevent expression of FABP4 in chin-APs (Fig. 1R). Note that *C/EBPα* expression was more sensitive to PIs treatment in chin and knee APs than *PPARγ* (Fig. 1J, K and P, Q), while these two genes were affected in a similar manner in hMADS cells (Fig. 1D,E).

All together these results indicated that depending on their origins, the different APs exhibit distinct sensitivities to the inhibitory effect of DRV or LPV on differentiation that may reflect the discrepancies observed in the antiretroviral-induced lipodystrophies.

### Lopinavir but not darunavir, altered adipose progenitors proliferation

As a decrease in differentiation alone is not sufficient to explain the unwanted side effects of PIs observed in the face of patients treated with PIs, we analyzed the effects of PIs on APs self-renewal.

**Figure 2:**
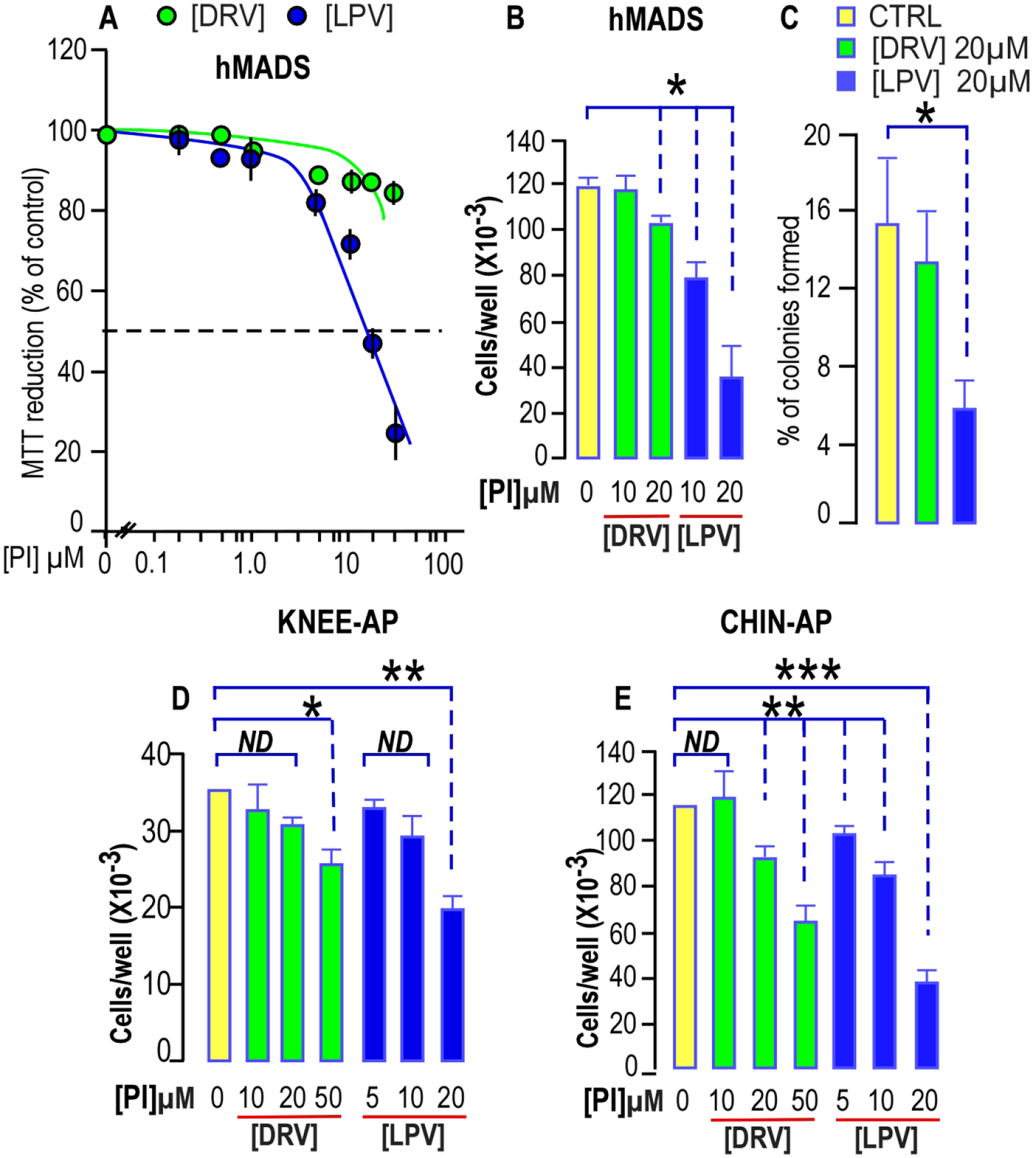
PIs impair proliferation and clonogenicity of hMADS cells, chin and Knee APs. (**A**) Dose response curve for DRV and LPV effect on hMADS cell proliferation. Proliferation was evaluated using the soluble tetrazolium salt MTT reduction assay after 4 days of proliferation in presence of DRV (green dots) or LPV (Blue dots). The results represent the mean ± SEM of three experiments carried out in triplicate. Error bars were omitted when the SEM was smaller than the size of the symbol. (**B**) Dose dependent effects of DRV and LPV on hMADS cell proliferation. Cells were seeded in 12-well plates and grown for 5 days in complete culture medium and counted using a BeckmanZ1 coulter. The results represent the mean ± SEM of three experiments carried out in triplicate (*p < 0.05). (**C**) Lopinavir but not Darunavir reduces clonogenicity of hMADS cells. After plating and growing 200 cells for 3 weeks in complete culture medium, the percentage of colonies obtained from hMADS cells was calculated. Mean ± SEM was representative of three independent experiments (*p < 0.05). (**D**,**E**) Dose dependent effects of DRV and LPV on knee and chin APs cell proliferation respectively. Cells were seeded in 12-well plates and grown for 5 days in complete culture medium and counted using a BeckmanZ1 coulter. Experiment was carried out using APs derived from the same person. The results represent the mean ± SEM of three experiments carried out in triplicate (*p < 0.05, **p < 0.01, ***p < 0.001). ND means “no significant difference with the control condition”.

We first treated hMADS cells with LPV or DRV for 4 days (MTT assay) or 5 days (cell counts) to assess their impact on proliferation. While increasing doses of DRV did not cause a huge decrease in the cell number, LPV treatment significantly reduced proliferation by 25 to 75% for concentrations starting from 10 µM (Fig. 2A and B).

In addition LPV dramatically reduced their ability to grow at a single cell level while DRV did not display any effect. All together these results indicate that LPV, but not DRV, alters self-renewal of hMADS cells (Fig. 2C).

These results prompted us to analyze self-renewal of APs isolated from different sites of the same donor upon PIs treatment. APs derived from chin were significantly impacted mainly by LPV while those derived from knee were less sensitive to PIs treatment (Fig. 2D and E). These results were confirmed after measuring the effects of PIs on proliferation of chin and knee APs both coming from another distinct donor (Supplementary Fig. 2).

We investigated the cytotoxic effects of PIs on APs, as we previously showed that low doses of LPV (1 µM) induced apoptosis in cancer stem cells expressing an embryonic signature^25^. After four days of culture in presence of LPV or DRV, cells were well attached to the tissue culture dish and more than 99.5% of cells were excluding trypan blue. We did not notice any induction of apoptosis by means of caspase 3 cleavage (Supplementary Fig. 3) indicating that the impairment of APs proliferation induced by HIV-PI did not result from cytotoxic effects.

### Signaling pathways altered by PIs

####  HIV-PIs’ effect on IER3 mRNA expression

**Figure 3:**
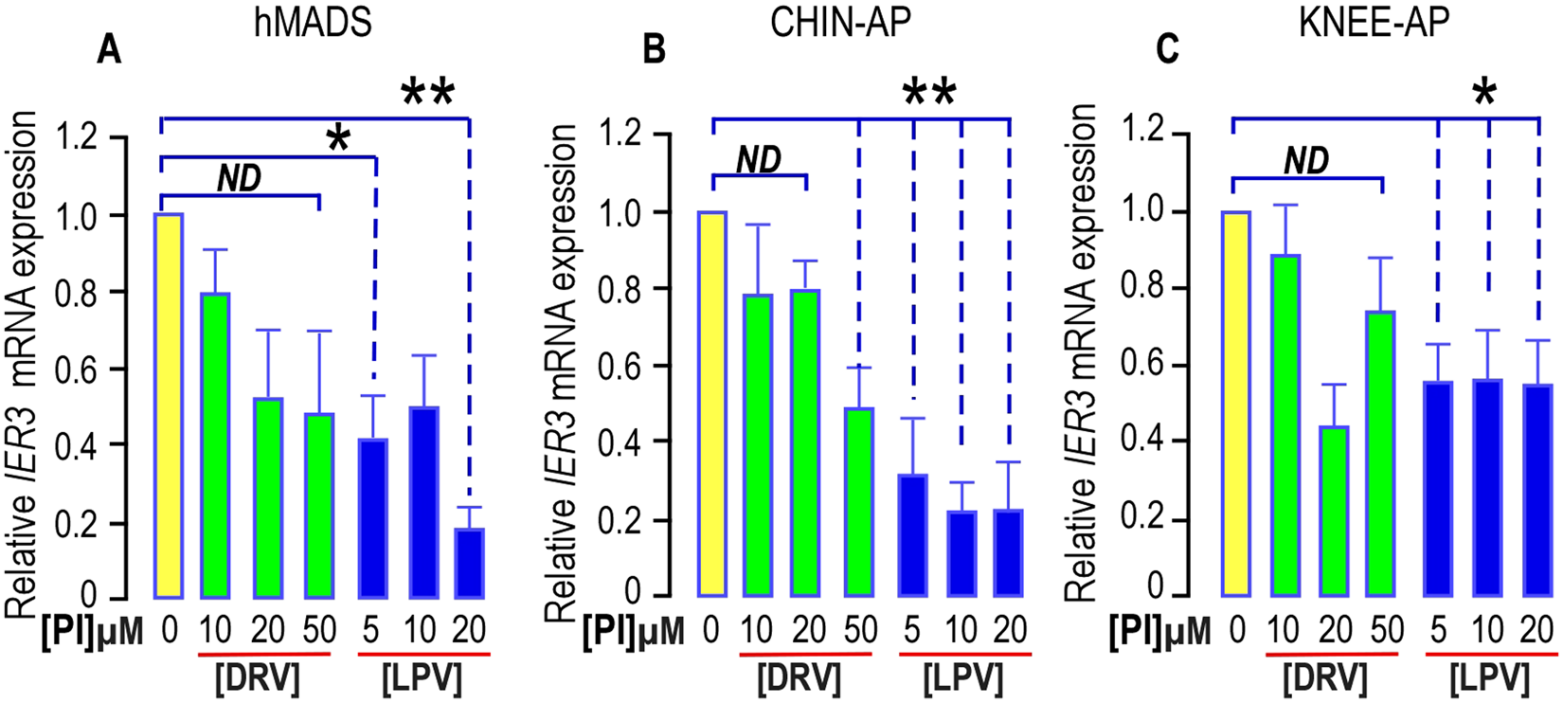
PIs impair IER3 expression in hMADS cells, chin and Knee APs. *IER3* expression was determined in APs submitted or not to increasing doses of DRV (green bars) or LPV (blue bars) for 5 days. The analysis findings were assessed by real-time RT-PCR and normalized for the expression of *36B4* mRNA. Mean ± SEM. was representative of three independent experiments. (*p < 0.05 and **p < 0.01). ND means “no significant difference”.

To gain mechanistic insights on PIs’ effect, we analyzed *IER3* expression in cells submitted to PIs treatment. In good correlation with the absence of inhibition of proliferation, a 5-day treatment with low doses of DRV did not impact significantly *IER3* expression either in hMADS cells or in the different APs used (Fig. 3A–C). In contrast LPV, which altered dramatically self-renewal, reduced *IER3* by 80% in hMADS cells and in chin APs (Fig. 3A and B). Knee APs were less sensitive as *IER3* expression was only reduced by 40% (Fig. 3C).

**Figure 4:**
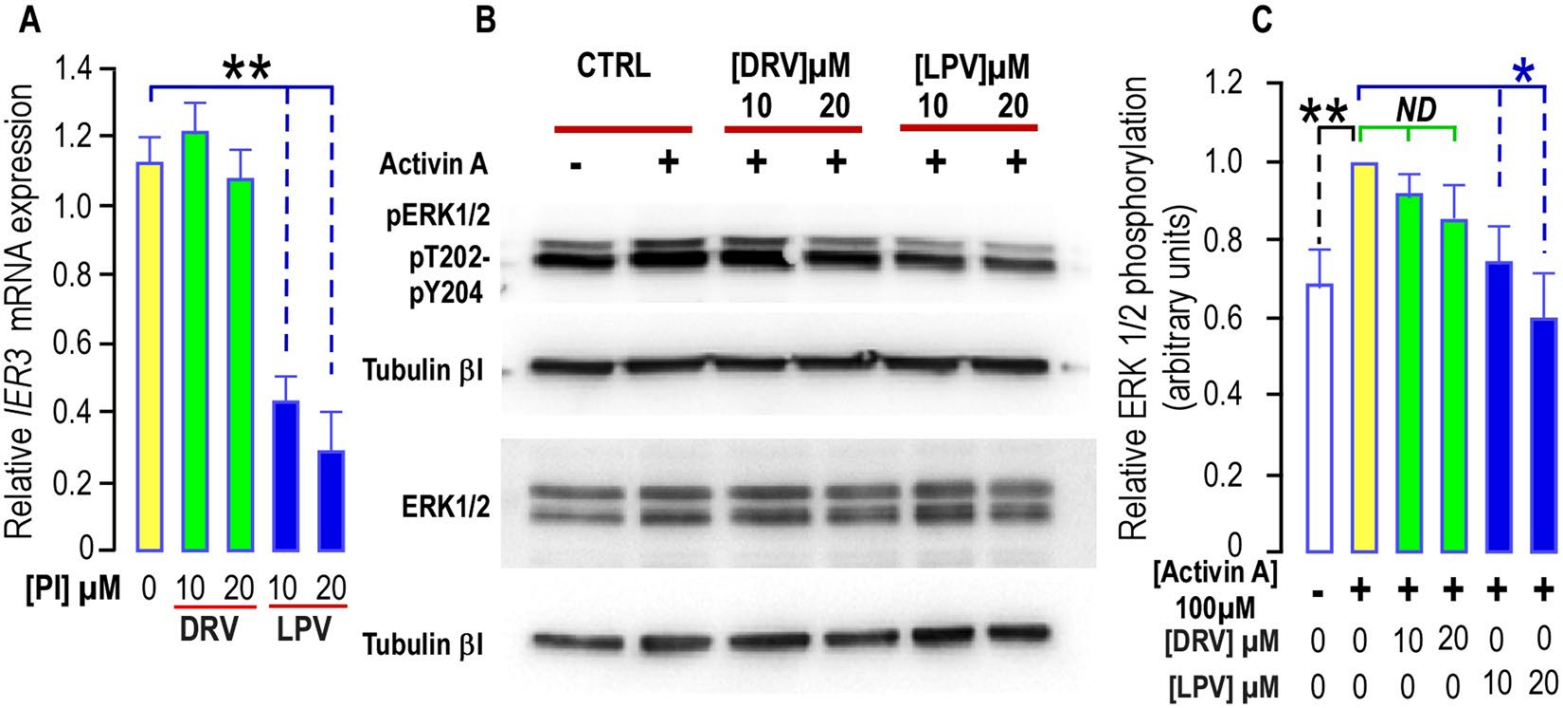
Long term treatment with PIs impairs *IER3* expression and ERK1/2 phosphorylation in hMADS cells. (**A**) Effect of PIs on *IER3* expression. *IER3* expression was determined in hMADS cells submitted or not to increasing doses of DRV (green bars) or LPV (blue bars) for 35 days. The analysis findings were assessed by real-time RT-PCR and normalized for the expression of *36B4* mRNA. Mean ± SEM was representative of three independent experiments. (*p < 0.05 and **p < 0.01). (**B**) PIs alter Activin A -induced ERK1/2 phosphorylation. Thirty µg of proteins prepared from hMADS cells treated with DRV or LPV for 35 days were loaded onto a 10% acrylamide gel. Sub-confluent cells were stimulated or not with 100 ng/ml activin A after 3 days of serum deprivation. Total or phosphorylated forms of ERK1/2 were assessed with specific antibodies. Activin A induced an increased phosphorylation of ERK1/2 that was blunted in cells treated with LPV. Expression of Tubulin-βI used as a loading control (lower panel). A representative Western blot is shown. (**C**) Quantification of the signals. The activin A-induced phosphorylation of ERK1/2 was measured using the Quantity one Program and compared to the expression of Tubulin-βI. Four and six determinations were analyzed when cells were treated respectively with DRV and LPV. Analysis was performed taking the unstimulated control as reference (black stars) or the activin A stimulated condition (green and blue stars) (*p < 0.05 and **p < 0.01). ND means “no significant difference”.

We analyzed IER3 expression after a longer chronic treatment of APs with PIs, as short term outcomes are not always reflecting the changes occurring in life-long cures. Indeed this decrease in *IER3* expression was still observed and was significant when a 35-day treatment was achieved in hMADS cells (Fig. 4A).

This indicated that concomitantly with APs proliferation, LPV impaired *IER3* mRNA expression. Its effects depended on the original location of the distinct APs.

#### ERK activation was blunted by LPV

We next examined the effects of PIs on signaling pathways important for cell proliferation in hMADS cells grown for 35-days in presence or absence of PIs. We measured the ERK1/2 phosphorylation in response to activin A which is essential to maintain APs (hMADS) self-renewal^7, 8^.

While DRV did not significantly affect ERK 1/2 phoshorylation in response to activin A, LPV dose-dependently blunted the phosphorylation of ERK1/2 observed in response to this factor (Fig. 4B and C). In presence of LPV, we measured a phosphorylation level that was not significantly different from the non-stimulated control level (Fig. 4C).

Neither DRV, nor LPV affected the activin A-induced SMAD2 phosphorylation indicating that this pathway leading to inhibition of differentiation through a decrease in CEBPβ-LAP expression is not targeted by PIs (Supplementary Fig. 4).

#### HIV-PIs modified the immediate early gene *EGR1* expression: consequences for *INHBA* gene expression

**Figure 5:**
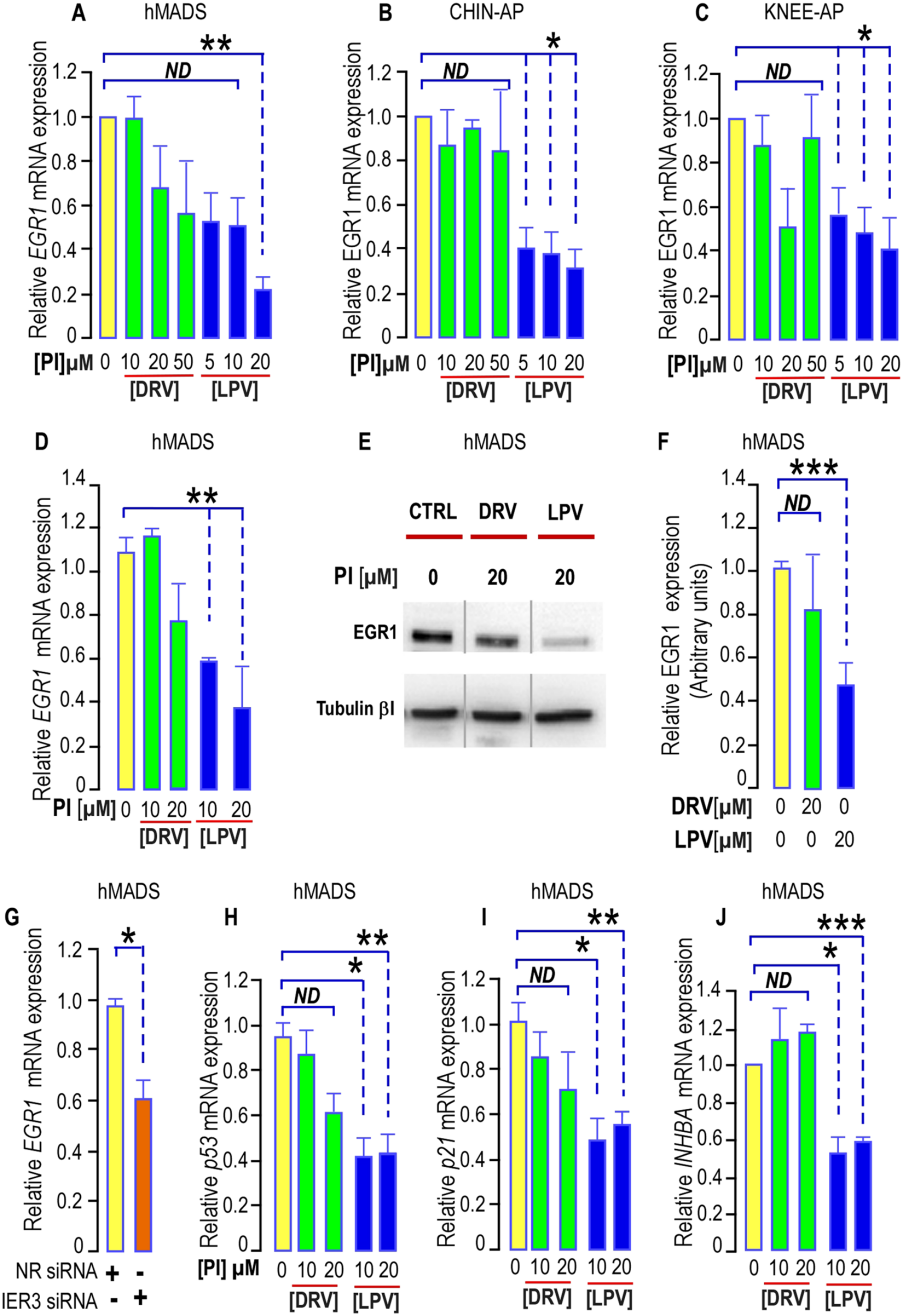
PIs impair EGR1 expression in hMADS cells, chin and Knee APs. (**A**–**D**) Effect of PIs on *EGR1* expression. (**A**–**C**) *EGR1* expression was determined in APs submitted or not to increasing doses of DRV (green bars) or LPV (blue bars) for 5 days. The analysis findings were assessed by real-time RT-PCR and normalized for the expression of *36B4* mRNA. Mean ± SEM. was representative of three independent experiments. (*p < 0.05 and **p < 0.01). ND means “no significant difference”. (**D**) *EGR1* expression was determined in hMADS cells submitted or not to increasing doses of DRV (green bars) or LPV (blue bars) for 35 days. The analysis findings were assessed by real-time RT-PCR and normalized for the expression of *36B4* mRNA. Mean ± SEM. was representative of three independent experiments. (*p < 0.05 and **p < 0.01). (**E**) LPV decreases EGR1 expression. Thirty µg of proteins prepared from hMADS cells grown for 35 days with DRV or LPV were loaded onto a 10% acrylamide gel. A representative Western blot showing the expression of Tubulin-βI used as a loading control (lower panel) and of EGR1 (upper panel) is presented. All bands are issued from the same exposure of the blots. (**F**) Quantification of the signals. EGR1 expression was measured using the Quantity one Program and compared to the expression of Tubulin-βI. Four independent determinations were analyzed (***p < 0.001). ND means “no significant difference”. (**G**) *EGR1* expression was determined in hMADS cells transfected with non-relevant siRNA or with siRNA to decrease IER3 expression for 3 days. The analysis findings were assessed by real-time RT-PCR and normalized for the expression of *36B4* mRNA. Mean ± SEM. was representative of four independent experiments (p = 0.0107). (*p < 0.05). (**H**,**I**,**J**) *Expression of EGR1 downstream genes*. *p53*, *p21 and INHBA* expressions were determined in hMADS cells submitted or not to increasing doses of DRV (green bars) or LPV (blue bars) for 35 days. The analysis findings were assessed by real-time RT-PCR and normalized for the expression of *36B4* mRNA. Mean ± SEM. was representative of three independent experiments (ND = not significantly different, *p < 0.05, **p < 0.01, ***p < 0.001).

To assess the consequences of the LPV-induced limitation in ERK1/2 phosphorylation on the ERK cascade, we measured *EGR1* expression in cells that received or not PIs treatment. We observed that neither a 5-day, nor a 35-day treatment with different concentrations of DRV significantly modified *EGR1* expression (Fig. 5A–D). In contrast, LPV strongly and significantly impaired *EGR1* expression irrespective of the length of the treatment (Fig. 5A–D). Inhibition was already reaching the maximum value at 5 µM LPV in chin-APs. A significant decrease was also observed at the protein level after 35 days of treatment with a 20 µM LPV concentration (Fig. 5E and F).

In addition, from RNA interference experiments with siRNAs to *IER3* that were previously validated^7^, we observed that *EGR1* expression was dependent on *IER3* expression (Fig. 5G), indicating that LPV may alter *IER3* prior to *EGR1*.

We measured the expression of *p53* which is directly controlled by *EGR1* at the transcriptional level^26^ and of its direct target *p21*. We found that the LPV-induced decrease in *EGR1* expression after 35 days of treatment was accompanied by a significant reduction in the mRNA levels of both *p53* and *p21* (Fig. 5H and I). DRV which does not modify EGR1 expression did not affect either the expression of *p53* or *p21*. This indicates that the decrease in *EGR1* expression is followed by functional consequences on the gene expression pattern of LPV-treated cells. Although a decreased expression of *p53* may favor cell growth, we did not observe such an event in LPV-treated cells.

In this regard, we focused on pathways important to sustain self-renewal in APs. *In silico* analysis revealed the presence of EGR1 binding elements in the *INHBA* promoter. We further measured the effect of LPV on *INHBA* expression. After 35 days of treatment with PIs, we observed that LPV decreased significantly *INHBA* expression in hMADS cells while DRV had no effect (Fig. 5J).

**Figure 6:**
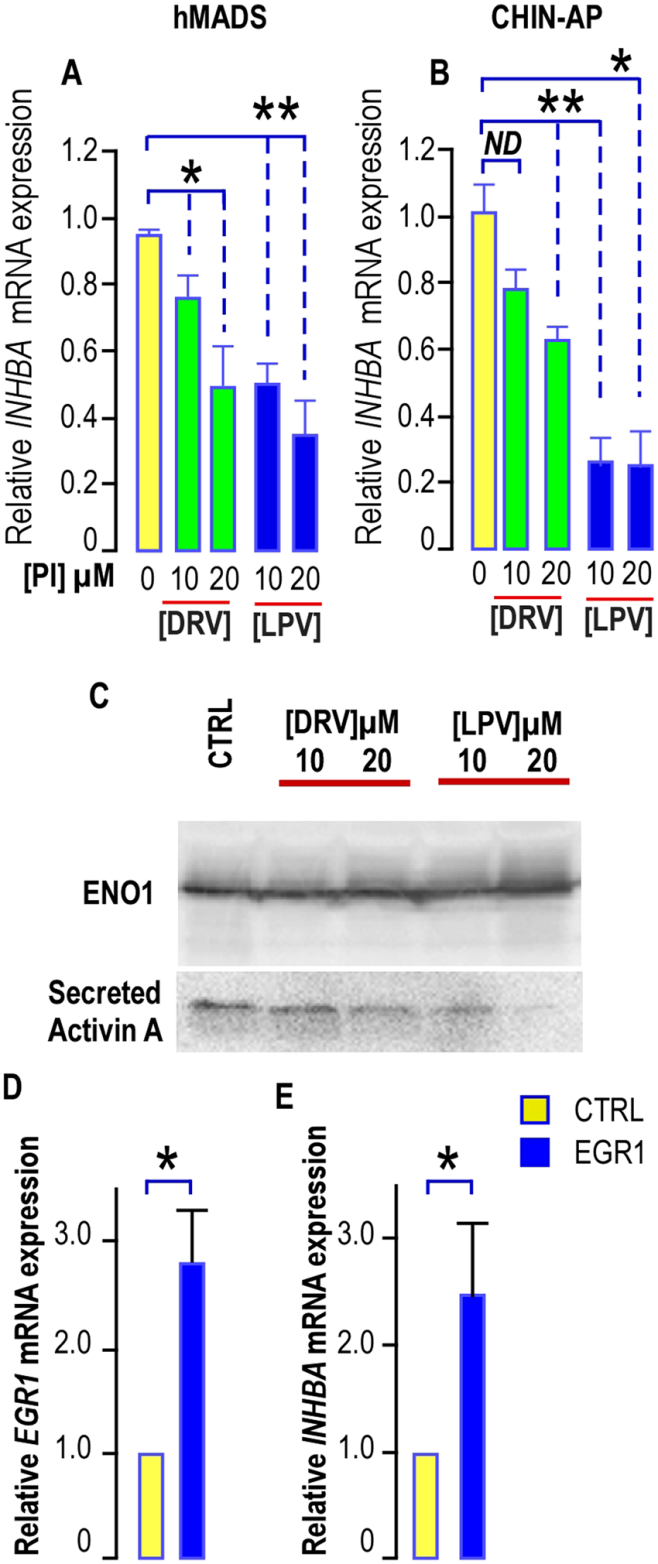
LPV impairs *INHBA* expression and activin A secretion. (**A**,**B**) *Expression of INHBA in hMADS cells and in chin-AP treated with PIs*. *INHBA* expression was determined in hMADS cells and chin APs submitted or not to increasing doses of DRV (green bars) or LPV (blue bars) for 5 days. The analysis findings were assessed by real-time RT-PCR and normalized for the expression of *36B4* mRNA. Mean ± SEM was representative of four (hMADS) or three (chin-AP) independent experiments (ND = not significantly different, *p < 0.05, **p < 0.01, ***p < 0.001). (**C**) *Effects of HIV-PIs on activin A secretion*. Culture media were collected after a 4-day treatment of hMADS cells with or without PIs, filtered on 0.45 µM membranes, and concentrated with Amicon ultra-15 columns (NMWL, three KDa; Millipore). Activin A secretion was analyzed by Western blot performed under non reducing conditions because anti–activin A antibody selectively binds to the dimeric form of activin A. A representative Western blot showing the expression of Enolase 1 (ENO1) used as a loading control (upper panel) and of activin A (Lower panel) is presented. (**D**,**E**) *Effect of EGR1 overexpression on INHBA*. *EGR1 and INHBA* expressions were determined in hMADS cells transduced with a lentivirus allowing *EGR1* expression. The analysis findings were assessed by real-time RT-PCR and normalized for the expression of *36B4* mRNA. Mean ± SEM was representative of four independent experiments. (*p < 0.05)

This observation prompted us to quantify *INHBA* expression in cells treated with PIs for a shorter period- i. e. 5 days. We observed that a short term treatment with LPV was sufficient to reduce *INHBA* expression in hMADS cells and in chin AP (Fig. 6A and B). To confirm these observations, we measured activin A secretion in PI-treated cells. Indeed we noticed that a four-day treatment with LPV reduced activin A secretion (Fig. 6C). All together, our results indicated that LPV altered self-renewal by both decreasing the production of activin A and impairing its signaling pathway, hindering then the autocrine loop sustaining the AP pool.

To ensure that EGR1 was involved in this regulation, *INHBA* expression was measured in cells overexpressing *EGR1* after lentiviral transduction. Indeed, we observed that a 3-time increase in *EGR1* expression led to an enhanced expression of *INHBA* (Fig. 6D and E) indicating that expression of *INHBA* was dependent on the presence of EGR1.

**Figure 7:**
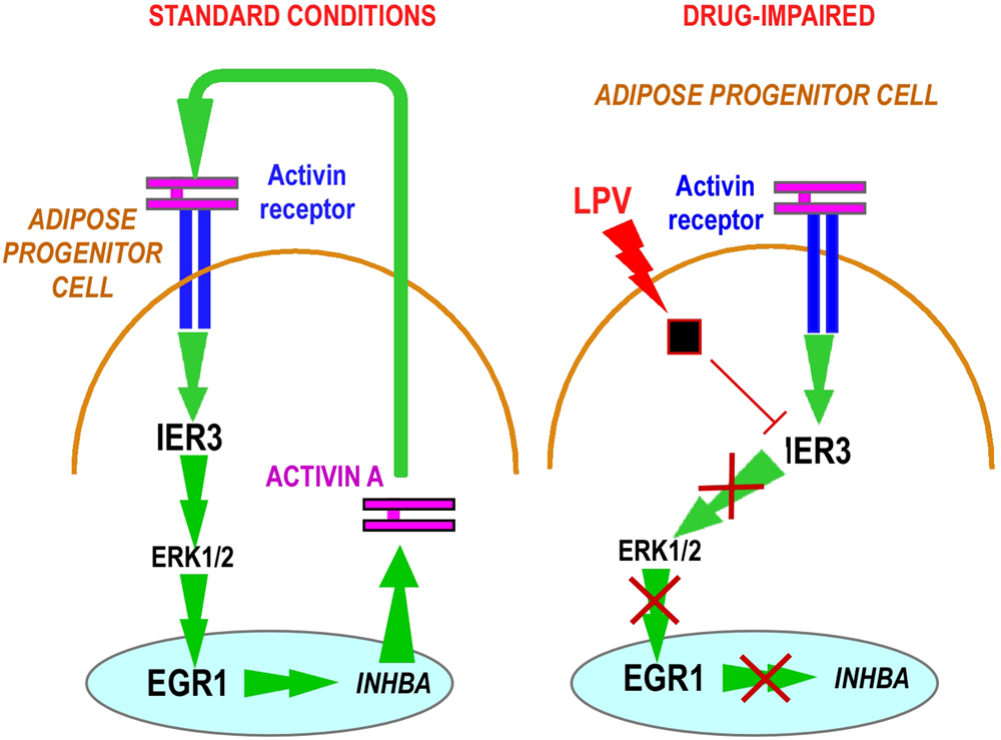
: Summary of the results found. Activin A sustains self-renewal through activation of IER3 expression. LPV impairs IER3 expression that impacts ERK1/2 phosphorylation and EGR1 expression. EGR1 downstream pathway is then affected. This implies a decrease in Activin A production and an impairment of the activin A autocrine signaling that alters proliferation and clonal expansion of APs.

The summary of the results and the pathway altered by HIV-PIs is presented in Fig. 7.

## Discussion

APs isolated from different depots display distinct molecular signatures which have been related to their embryonic origin^5, 6^. In this paper we aimed to point out the heterogeneity of APs derived from human distinct fat depots by means of their respective sensitivities to pharmaceutical treatments. We focused on drugs known to affect adipose tissue homeostasis: the PIs which are part of HIV–ART therapy. Among its deleterious side effects, this treatment influences adipose tissues repartition. It promotes lipodystrophy development, leading then to a paucity of fat that impairs energy homeostasis. This represents a relevant approach to pinpoint the specific features (self-renewal and differentiation) of distinct APs and to identify the pathways playing a crucial role in their perpetuation.

We used APs from different origins^6^ in addition to abundantly described models of APs^17, 18^. Ten years ago, the inhibition of adipose differentiation induced by PIs and NRTI was reported in murine as well as human models^17^. A lack of differentiation was the only designed culprit responsible for lipodystrophies -even lipoatrophies- and this mechanism appeared as sufficient to explain the evolution of this unwanted side effect. In good agreement with these results, we found here that adipose differentiation of hMADS and APs from knee was drastically impacted by LPV treatment in a dose-dependent manner. However, the chin-derived APs were only impacted significantly when 20 µM LPV was used, thus pointing out important differences in sensitivity to LPV treatment among the distinct AP pools. This indicated that inhibition of differentiation may not be the major cause of lipoatrophy in the chin fat pad induced by HIV-ART. Indeed we found that proliferation of chin APs was dramatically reduced both by LPV and DRV, LPV being the most efficient molecule for doses as low as 5 µM significantly impaired cell growth. Note that these molecules did not display cytotoxic effects in APs while low doses of LPV induced apoptosis in cancer stem cells expressing an embryonic signature^25^, reflecting again huge differences in sensitivity between distinct cell types. In this regard, APs from different depots displayed proper sensitivities to DRV and LPV. We measured this effect in paired samples issued from different donors indicating that it was an unrestricted observation. Proliferation was also reduced in the other APs analyzed here, and this effect has to be considered in addition to the inhibition of differentiation, indicating that several processes may contribute all together to alteration of the fat pads expansion. Absence of differentiation efficiently reduces production of adipocytes when APs are present. In addition any reduction in the AP pool is susceptible to cause adipose tissue remodeling and to decrease fat depot development.

Our *in vitro* observations are in lane with clinical reports revealing that different manifestations of the HIV-ART were observed in AIDS patients. As an example, lipoatrophy of the face and neck adipose tissues can be observed concomitantly with hypertrophy of the visceral adipose tissue. These two antagonistic situations could even be observed in the same patient indicating that all adipose depots do not display identical responses to HIV-PI *in vivo* ^16^. Of note, PIs were reported as lipoatrophy-inducers in the face^27^. Thus, any shrinkage of the AP pool may account for the irreversible HIV-ART-induced lipoatrophies that were observed.

We then aimed to decipher the molecular mechanisms altered by HIV-ART that underlie the adverse effects on APs self-renewal.

Several genes and signaling pathways control the balance between self-renewal and differentiation in stem cells. Recently, we focused on activin A and its crucial role to maintain self-renewal in adipose progenitors^7, 8^. We pointed out that activin A was involved in the preservation of AP pools by coordinating the balance between self-renewal and differentiation in an autocrine manner^7, 8^. Similarly, activin A also sustains self-renewal of human ES cells^28, 29^ and its upregulation is required to maintain mesenchymal features of cancer stem-like cells in non-small cell lung cancer^30^ indicating that its role is not restricted to APs.

The immediate early response gene *IER3* is expressed in a wide range of tissues^31^ and we recently showed that activin A controls its expression^7^. It has been reported to participate to several processes including cell survival, proliferation or apoptosis^9, 32^. Its pro-survival properties are relevant in a context of stem cells that need to perpetuate all life-long but also in a context of a tissue that needs to adapt to metabolic constraints^33–35^. Indeed, expression of *IER3* was crucial to maintain and expand the adipose progenitor pool in response to effectors present in the microenvironment, including activin A^7^. Although, an elevation of *IER3* expression was measured in the APs isolated from obese patients, information concerning its expression in lipodystrophic individuals is scant and difficult to reach due to ethical considerations. We noted that LPV treatment which altered self-renewal of APs lowered *IER3* expression in these cells. This indicated that this gene is closely linked to the self-renewal status of the AP pools. Interestingly, not all the APs responded in a similar manner. We observed a consequent LPV-induced diminution in *IER3* expression mainly in chin-derived APs, which is in lane with the LPV-induced alteration of proliferation measured in these cells.

From RNA interference experiments, we previously showed that IER3 supports activin A-induced self-renewal of APs through phosphorylation of ERK1/2 and activation of this regulatory network^7^. In good agreement, we observed that LPV, which reduces *IER3* expression, significantly blunted the activin A–induced phosphorylation of ERK1/2 in APs. The alteration of this key pathway involved in cell proliferation may account for the negative impact of LPV on proliferation. In addition, these results and those previously reported^7^ show that *IER3* expression is correlated with the expandability of the adipose tissue and reflects its adaptation to metabolic constraints in physiopathological conditions.

To further identify the cascade leading to a decrease in proliferation, we concentrated on the multifunctional transcription factor EGR1 which is highly dependent on ERK1/2 phosphorylation in self-renewing stem cells^36, 37^. Furthermore, *EGR1* has been identified as one of the positively regulated target genes by activin A in the murine gonadotrope-derived LBT2 cell line^38^. EGR1 is a DNA binding protein able to regulate the expression of a large number of genes, then controlling important cellular processes such as survival, cell growth, differentiation, senescence, transformation or apoptosis. In addition to the three zinc finger motifs and two nuclear localization signals, the structural analysis reveal the presence of a strong activation followed by a repression domain while a weak activation domain is located at the C terminus, indicating that EGR1 can function as a positive or negative regulator of transcription^39^. For instance, EGR1 promotes cell proliferation in some cancers cells^40, 41^ and has also been associated with enhanced cell survival and tumor progression^20, 42^, while inhibition of EGR1 contributes to anti mitogenic effects in vascular smooth muscle cells^43^ or cancer cells^44^. EGR1 was shown to play an essential role in many differentiation processes leading to the production of functional cell types including B cells, macrophages, neuroblastoma cells and adipocytes^45^. Its anti-adipogenic action is important to maintain appropriate levels of adipogenesis and/or a pool of resting APs able to undergo specialization upon appropriate stimulus in their microenvironment. In agreement with this finding, RNA interference experiments revealed that *EGR1* expression was depending on *IER3*, an important target in the activin A self-renewal signaling pathway in our model. Our results indicated that LPV negatively targeted EGR1 expression as well as the expression of its downstream genes including *INHBA*, while *EGR1* overexpression promoted *INHBA* expression. All together, these observations are in lane with a LPV-induced alteration of self-renewal of APs and they bring further evidences in the identification of the actors involved in the activin A autocrine loop that sustains APs self-renewal.

So far, the intracellular receptor or target for LPV remains unknown. Our results indicate that LPV, but not DRV, blunts the proliferation/self-renewal pathway activated by activin A. Note that LPV reduces also its production by lowering the transcription of *INHBA* gene and the secretion of activin A in APs. This contributes to turn down one of the autocrine loops critical to maintain the stemness properties of the AP pool. Note that any deterioration of this pathway is sufficient to dramatically alter self-renewal.

In conclusion, our work underlines the heterogeneity in the responses of distinct APs to HIV-PIs. While proliferation and self-renewal were preferentially impaired in APs derived from chin, differentiation was mostly impacted in APs derived from knee, indicating that all features of APs may account for lipodystrophy development. We identified a cascade of events that were important to sustain the self-renewal ability of APs. Cell autonomous regulation through activin A production appeared as an important, and somehow underestimated so far, pathway to perpetuate the AP pools. In this regard, the identification of *IER3* and *EGR1* as crucial genes to control self-renewal in physio-pathological conditions may give new insights to target and control the adipose tissue development and indeed for successful *de novo* adipogenesis in restoring facial defects.

## Material and Methods

### Reagents

Unless specified otherwise, all reagents were obtained from Sigma (Saint-Quentin Fallavier, France).

Tissue culture media were obtained from LONZA (Levallois-Perret, France) and foetal calf serum (FCS) from Dutscher S.A. (Brumath, France). Protease Inhibitors were obtained by extraction from commercially available tablets and capsules and purified by silica gel column chromatography. Their purity was assessed by ^1^H and ^13^C nuclear magnetic resonance and mass spectroscopy. Pure molecules were dissolved in DMSO. The key intermediates used for structure-activity relationship studies were synthesized according to a previously reported procedure^46^.

### Cell culture

Human APs cells used for *in vitro* studies derived from distinct sources.

hMADS cells^6^ were isolated more than fifteen years ago from adipose tissue, as surgical scraps from surgical specimen of various surgeries of young donors, with the informed consent of the parents. All methods were approved and performed in accordance with the guidelines and regulations of the Centre Hospitalier Universitaire de Nice Review Board.

Chin and knee paired APs were derived from the stroma vascular fraction of Caucasians women who underwent elective liposuction procedures. Informed consent was obtained from all patients^44, 45^. APs were grown, maintained and differentiated according to experimental conditions previously described for each cell type^6, 45, 47^.

#### For proliferation experiments

cells were seeded at 3 000 cells/cm^2^. They were incubated in complete culture medium. The cells were counted using a Beckman Coulter Z1.

#### Proliferation based on MTT assay

Proliferation of human APs was evaluated using the soluble tetrazolium salt MTT reduction assay^48^. Briefly, cells were seeded at 1500 cells/well in quadruplicate in 96-well plates and incubated at 37 °C for the indicated time. MTT (0.5 mg/ml) was added to each well and cells were further incubated for 4 h. Then the insoluble formazan was dissolved in isopropanol and DMSO (v/v) and the OD was determined spectrophometrically at 562 nm and 630 nm for background correction.

#### A clonogenic assay

was carried out by plating 200 cells in a 100 mm-dia. dish in complete growth medium (containing 10% SVF) for 21 days. Colonies were scored after staining with crystal violet (1%). Each experiment was carried out in triplicate.

#### Adipocytic differentiation

was carried out as previously described and was assessed by Oil Red O staining^6, 47^.

### Viral infection

Lentiviral particles were produced transfecting the 293 T cell line with the 2K7BSD-EGR1 constructs along with the packaging vectors. After lentiviral infection, cell lines stably expressing EGR1 were selected in appropriate blasticidin containing- medium (1 *μ*g/mL) for at least 15 days. Two stable cell lines were developed from independent viral productions/infections and exhibited identical behaviors. Cells stably expressing the TOMATO-Fluorescent Protein (TOMATO) were obtained after 2K7BSD-Tomato lentiviral particles infection and were used as control.

### Gene expression analysis

Total RNA was extracted using the TRI-Reagent kit (Euromedex, Soufflweyersheim, France) and reverse transcription (RT) was performed using MMLV reverse transcriptase (Promega, Charbonnieres, France), as recommended by the manufacturers.

All primer sequences are described in supplementary section. Real-time PCR assays were run on an ABI Prism One step real-time PCR machine (Applied Biosystems, Courtaboeuf, France). Normalization was performed using *36B4* as a reference gene. Quantification was performed using the comparative Ct method.

### Protein expression

Cells were rinsed in ice-cold PBS and whole cell extracts were prepared as described^7^. Briefly, cells were lysed in the cell lysis buffer^49^, sonicated for 10 s and centrifuged at 12000 g for 10 min.

Thirty micro grams of proteins were resolved by SDS–PAGE under reducing conditions and transferred to Immobilon–P membranes (Millipore, Molshiem, France). The detection antibodies listed in supplementary section were used according to the manufacturer’s instructions.

The bound primary antibody was detected by horseradish peroxidase-conjugated secondary antibody and visualized using an ECL detection kit (Millipore, Molsheim, France).

Chemiluminescence was observed using a molecular imager ChemiDoc XRS system (Bio-Rad, Marne la Coquette, France). The band intensity was quantified using Bio-Rad Quantity One software.

### Activin A secretion

Activin A secretion was assessed as previously described^8^. Briefly, hMADS cells were maintained in 5 µg/ml insulin and 10 µg/ml transferrin medium in the absence or presence of LPV or DRV as indicated. Culture media were collected 4 days later, filtered on 0.45 µm membranes, and concentrated with Amicon ultra-15 columns (NMWL, three KDa; Millipore). Levels of activin A were analyzed by Western blot performed under nonreducing conditions because anti–activin A antibody selectively binds to the dimeric form of activin A. Secreted enolase one (ENO1) was used as a loading control^50^.

### Statistical analysis

The results are shown as mean ± standard error of the mean (SEM), with the number of experiments indicated. Statistical significance was determined by *t-*tests using Micrococal Origin 6.0 (Micrococal Software, Northampton MA). Probability values <0.05 were considered statistically significant and are marked with a single asterisk, <0.01 with double asterisks and <0.001 with triple asterisks.

## Electronic supplementary material

Supplementary Information

## References

[1] Enerback S (2010). Human brown adipose tissue. Cell Metab.

[2] Tchkonia T (2002). Fat depot origin affects adipogenesis in primary cultured and cloned human preadipocytes. Am J Physiol Regul Integr Comp Physiol.

[3] Tchkonia T (2005). Abundance of two human preadipocyte subtypes with distinct capacities for replication, adipogenesis, and apoptosis varies among fat depots. Am J Physiol Endocrinol Metab.

[4] Maumus M (2008). Evidence of *in situ* proliferation of adult adipose tissue-derived progenitor cells: influence of fat mass microenvironment and growth. J Clin Endocrinol Metab.

[5] Billon N (2007). The generation of adipocytes by the neural crest. Development.

[6] Kouidhi, M. *et al*. Characterization of human knee and chin adipose-derived stromal cells. *Stem Cells Int* **2015**, Epub 2015 Feb 2013 (2015). 10.1155/2015/592090 PMC4334981 25733979

[7] Ravaud C (2015). IER3 is a crucial effector promoting expansion of adipose progenitor cells in response to distinct micro-environmental effectors. Stem cells.

[8] Zaragosi LE (2010). Activin a plays a critical role in proliferation and differentiation of human adipose progenitors. Diabetes.

[9] Arlt A, Schafer H (2011). Role of the immediate early response 3 (IER3) gene in cellular stress response, inflammation and tumorigenesis. Eur J Cell Biol.

[10] Caron-Debarle M, Lagathu C, Boccara F, Vigouroux C, Capeau J (2010). HIV-associated lipodystrophy: from fat injury to premature aging. Trends Mol Med.

[11] Carr A, Cooper DA (2000). Adverse effects of antiretroviral therapy. Lancet.

[12] Nolis T (2014). Exploring the pathophysiology behind the more common genetic and acquired lipodystrophies. J Hum Genet.

[13] Huang-Doran I, Sleigh A, Rochford JJ, O’Rahilly S, Savage DB (2010). Lipodystrophy: metabolic insights from a rare disorder. J Endocrinol.

[14] Singhania R, Kotler DP (2011). Lipodystrophy in HIV patients: its challenges and management approaches. HIV AIDS (Auckl).

[15] Alam N (2012). Body fat abnormality in HIV-infected children and adolescents living in Europe: prevalence and risk factors Fat abnormality in children. J Acquir Immune Defic Syndr.

[16] Giralt M, Domingo P, Villarroya F (2011). Adipose tissue biology and HIV-infection. Best Pract Res Clin Endocrinol Metab.

[17] Vernochet C (2005). Human immunodeficiency virus protease inhibitors accumulate into cultured human adipocytes and alter expression of adipocytokines. J Biol Chem.

[18] Rodriguez AM (2005). Transplantation of a multipotent cell population from human adipose tissue induces dystrophin expression in the immunocompetent mdx mouse. J Exp Med.

[19] Min IM (2008). The transcription factor EGR1 controls both the proliferation and localization of hematopoietic stem cells. Cell Stem Cell.

[20] Virolle T (2003). Egr1 promotes growth and survival of prostate cancer cells. Identification of novel Egr1 target genes. J Biol Chem.

[21] Thiel G, Cibelli G (2002). Regulation of life and death by the zinc finger transcription factor Egr-1. J Cell Physiol.

[22] Zwang Y (2011). Two phases of mitogenic signaling unveil roles for p53 and EGR1 in elimination of inconsistent growth signals. Mol Cell.

[23] Boffito M (2007). Pharmacokinetics and antiretroviral response to darunavir/ritonavir and etravirine combination in patients with high-level viral resistance. Aids.

[24] Jackson A (2011). Pharmacokinetics of plasma lopinavir/ritonavir following the administration of 400/100 mg, 200/150 mg and 200/50 mg twice daily in HIV-negative volunteers. J Antimicrob Chemother.

[25] Darini CY (2013). Targeting cancer stem cells expressing an embryonic signature with anti-proteases to decrease their tumor potential. Cell Death Dis.

[26] Nair P (1997). Early growth response-1-dependent apoptosis is mediated by p53. J Biol Chem.

[27] Carr A (1999). Diagnosis, prediction, and natural course of HIV-1 protease-inhibitor-associated lipodystrophy, hyperlipidaemia, and diabetes mellitus: a cohort study. Lancet.

[28] Vallier L, Alexander M, Pedersen RA (2005). Activin/Nodal and FGF pathways cooperate to maintain pluripotency of human embryonic stem cells. J Cell Sci.

[29] Xiao L, Yuan X, Sharkis SJ (2006). Activin A maintains self-renewal and regulates fibroblast growth factor, Wnt, and bone morphogenic protein pathways in human embryonic stem cells. Stem Cells.

[30] Wamsley JJ (2015). Activin upregulation by NF-kappaB is required to maintain mesenchymal features of cancer stem-like cells in non-small cell lung cancer. Cancer Res.

[31] Feldmann KA, Pittelkow MR, Roche PC, Kumar R, Grande JP (2001). Expression of an immediate early gene, IEX-1, in human tissues. Histochem Cell Biol.

[32] Shen L, Guo J, Santos-Berrios C, Wu MX (2006). Distinct domains for anti- and pro-apoptotic activities of IEX-1. J Biol Chem.

[33] Pawlikowska P (2009). ATM-dependent expression of IEX-1 controls nuclear accumulation of Mcl-1 and the DNA damage response. Cell Death Differ.

[34] Steensma DP (2009). Rearrangements and amplification of IER3 (IEX-1) represent a novel and recurrent molecular abnormality in myelodysplastic syndromes. Cancer Res.

[35] Trouillas M (2009). Three LIF-dependent signatures and gene clusters with atypical expression profiles, identified by transcriptome studies in mouse ES cells and early derivatives. BMC Genomics.

[36] Cook PJ (2016). Cox-2-derived PGE2 induces Id1-dependent radiation resistance and self-renewal in experimental glioblastoma. Neuro Oncol.

[37] Sakakini N (2016). A Positive Feed-forward Loop Associating EGR1 and PDGFA Promotes Proliferation and Self-renewal in Glioblastoma Stem Cells. J Biol Chem.

[38] Mazhawidza W, Winters SJ, Kaiser UB, Kakar SS (2006). Identification of gene networks modulated by activin in LbetaT2 cells using DNA microarray analysis. Histol Histopathol.

[39] Gashler AL, Swaminathan S, Sukhatme VP (1993). A novel repression module, an extensive activation domain, and a bipartite nuclear localization signal defined in the immediate-early transcription factor Egr-1. Mol Cell Biol.

[40] Baron V, Adamson ED, Calogero A, Ragona G, Mercola D (2006). The transcription factor Egr1 is a direct regulator of multiple tumor suppressors including TGFbeta1, PTEN, p53, and fibronectin. Cancer Gene Ther.

[41] Sun T, Tian H, Feng YG, Zhu YQ, Zhang WQ (2013). Egr-1 promotes cell proliferation and invasion by increasing beta-catenin expression in gastric cancer. Dig Dis Sci.

[42] Baron V (2003). Inhibition of Egr-1 expression reverses transformation of prostate cancer cells *in vitro* and *in vivo*. Oncogene.

[43] Kimura TE (2014). Inhibition of Egr1 expression underlies the anti-mitogenic effects of cAMP in vascular smooth muscle cells. J Mol Cell Cardiol.

[44] DeLigio JT, Zorio DA (2009). Early growth response 1 (EGR1): a gene with as many names as biological functions. Cancer Biol Ther.

[45] Boyle KB (2009). The transcription factors Egr1 and Egr2 have opposing influences on adipocyte differentiation. Cell Death Differ.

[46] Stoll V (2002). X-ray crystallographic structure of ABT-378 (lopinavir) bound to HIV-1 protease. Bioorg Med Chem.

[47] Rodriguez AM (2004). Adipocyte differentiation of multipotent cells established from human adipose tissue. Biochem Biophys Res Commun.

[48] Velasco-Velazquez MA (2003). 4-Hydroxycoumarin disorganizes the actin cytoskeleton in B16-F10 melanoma cells but not in B82 fibroblasts, decreasing their adhesion to extracellular matrix proteins and motility. Cancer Lett.

[49] Peraldi P, Xu M, Spiegelman BM (1997). Thiazolidinediones block tumor necrosis factor-alpha-induced inhibition of insulin signaling. J Clin Invest.

[50] Chiellini C (2008). Characterization of human mesenchymal stem cell secretome at early steps of adipocyte and osteoblast differentiation. BMC Mol Biol.

