# Supplementary material for: Impairment of the activin A autocrine loop by lopinavir reduces self-renewal of distinct human adipose progenitors
Source: Sci Rep. 2017 Jun 7;7:2986. doi: 10.1038/s41598-017-02807-9 (PMC5462747; doi:10.1038/s41598-017-02807-9)
Supplement: Supplementary file 1 — Supplementary Information [file 41598_2017_2807_MOESM1_ESM.pdf]

SUPPLEMENTARY INFORMATION

Impairment of the activin A autocrine loop by lopinavir reduces self-renewal of distinct human adipose progenitors.

Christophe RAVAUD [1], Martin PARE[1], Stéphane AZOULAY[2],

Christian DANI [1, 3] and Annie LADOUX [1,3]*

Supplementary Information

Sequences for the primers used in this study

**Gene**	**Forward primer**	**Reverse primer**
IER3	5'- AACCGAACCCAGCCAAAG -3',	5'- ACACCCTCTTCAGCCATCAG -3',
EGR1	5'- TGCTGGTGGAGACCAGTTAC-3'	5'- AAGCGGCCAGTATAGGTGAT-3'
36B4	5'-CTACAACCCTGAAGAAGTGCTTG-3'	5'- CAATCTGCAGACAGACACTGG-3'
C/EBPα	5'- CTTGTGCCTTGGAAATGCAAATGCAA-3'	5'- GCTGTAGCCTCGGGAAGGA-3'
p53	5'- CTACCAGGGCAGCTACGGTTT-3'	5'- CTGCACAGGGCAGGTCTTG-3'
p21	5'- AAGACCATGTGGACCTGT-3'	5'-GGTAGAAATCTGTCATGCTG-3'
INHBA	5'- GGGAGAACGGGTATGTGGAGAT-3'	5'-GCTGTTCCTGACTCGGCAAA-3'
PPARγ	***5'-***AGCCTCATGAAGAGCCTTCCA-3'	5'- TCCGGAAGAAACCCTTGCA-3'

siRNA sequences:

IER3 : 5'ACCUCAGCACUUUCCUCCAGCAACA3' (Invitrogen, Cergy-Pontoise, France)

Supplementary data: Table 1

ANTIBODY	MANUFACTURER	CATALOG NUMBER	DILUTION
ANTI-Phospho ERK 1/2	Cell Signaling Technology	#4370	2000
ANTI-ERK 1/2	Cell Signaling Technology	#9102	1000
ANTI-EGR1	Santa Cruz Biotechnology	#sc-189	500
Anti FABP4	Millipore	# MABS172	1000
ANTI-phospho Smad2	Cell Signaling Technology	#3104	1000
ANTI-CASPASE 3	Cell Signaling Technology	#9664	1000
ANTI-CLEAVED CASPASE 3	Cell Signaling Technology	#9665	500
ANTI-Activin A	R&D Systems	#MAB 3381	500
ANTI-α enolase	Santa Cruz Biotechnology	#SC-1543	1000
Anti FABP4	Millipore	# MABS172	1000

References for the antibodies used in this study.

Legends for Supplementary figures

Supplementary Figure 1:

Adipocyte differentiation in hMADS, knee or chin APs.

After induction of differentiation for 17 days in absence or presence of PIs at the indicated concentrations, cells were fixed and further stained with OilRed O. Differentiated cells displayed red staining within the lipid droplets. The panels are issued from the same experiments presented in Fig. 1, with higher magnification. The scale represents 100µM.

Supplementary Figure 2: Dose dependent effects of DRV and LPV on knee and chin APs cell proliferation respectively.

Cells were seeded in 12-well plates and grown for 5 days in complete culture medium and counted using a BeckmanZ1 coulter. Experiment was carried out using APs derived from the same person. The results represent the mean ± SEM of three experiments carried out in triplicate (** p<0.01, *** p<0.001). ND means "no significant difference with the control condition".

Supplementary Figure 3:

LPV and DRV do not exert cytotoxic effects on adipose progenitors.

Analysis of apoptosis: Western blot analysis of caspase-3 cleavage in APs grown for 5 days in absence or presence of increasing concentrations of DRV and LPV. Tubulin is shown as a loading control. These blots are representative of two independent experiments made in duplicate for both cell types. The control condition corresponds to cells that did not receive DRV or LPV treatment. Positive control for caspase 3 cleavage corresponds to cells incubated overnight with puromycin (4µg/ml) or staurosporine (2µM). No caspase 3 cleavage was detected in control and HIV-PI treated cells. Representative Western blots are shown

Supplementary Figure 4

PIs do not alter activin A -induced phosphorylation of SMAD 2

Thirty µg of proteins prepared from hMADS cells treated with DRV or LPV for 35 days were loaded onto a 10% acrylamide gel. Sub-confluent cells were stimulated or not with 100 ng/ml activin A after 3 days of serum deprivation. Phosphorylated forms of SMAD 2 were assessed with specific antibodies. Activin A stimulation induced increased phosphorylation of SMAD2 in control cells and in cells treated with PIs. Expression of Tubulin-βI used as a loading control (lower panel). A representative Western blot is shown.

SUPPLEMENTARY FIGURE 1

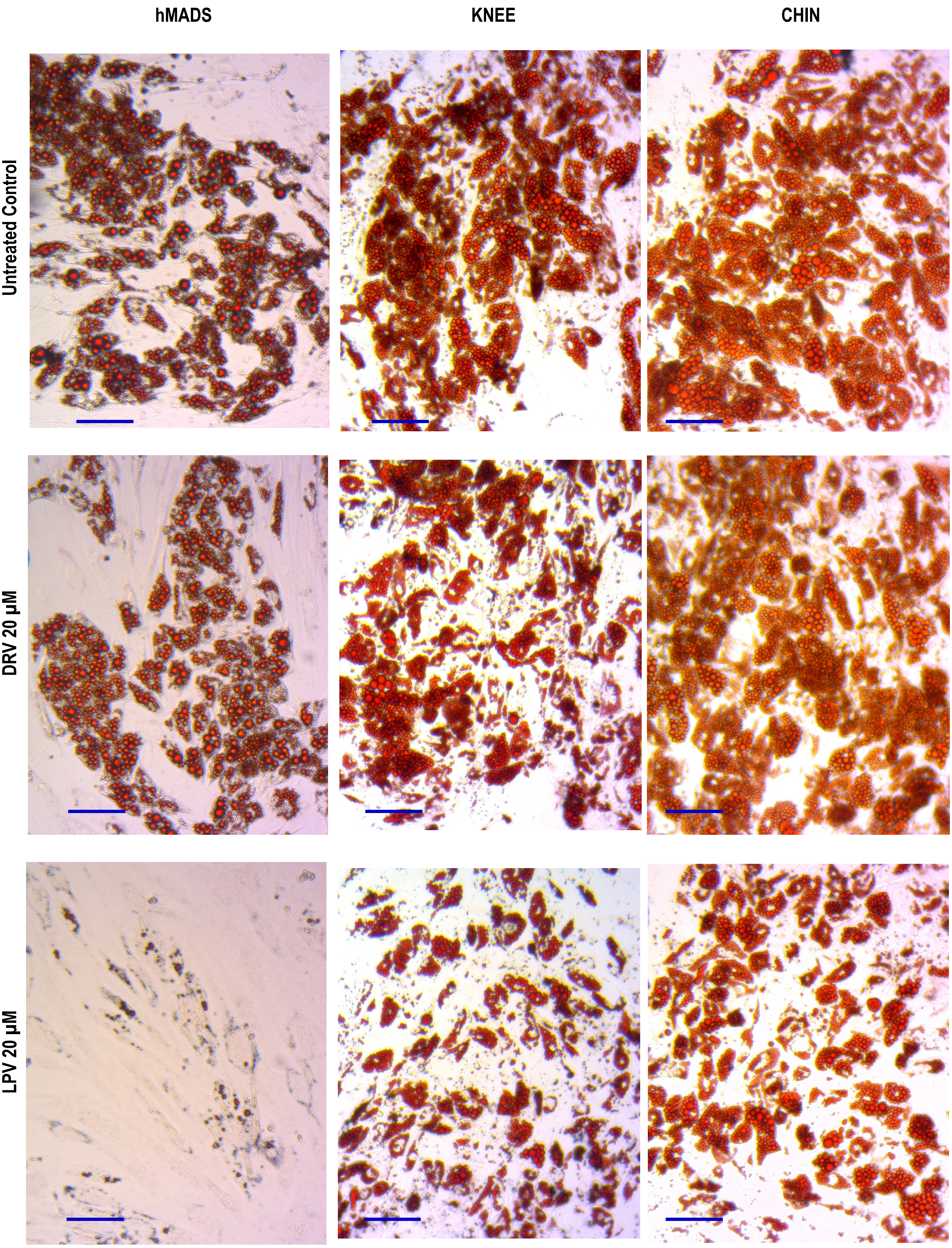

Supplementary figure 2

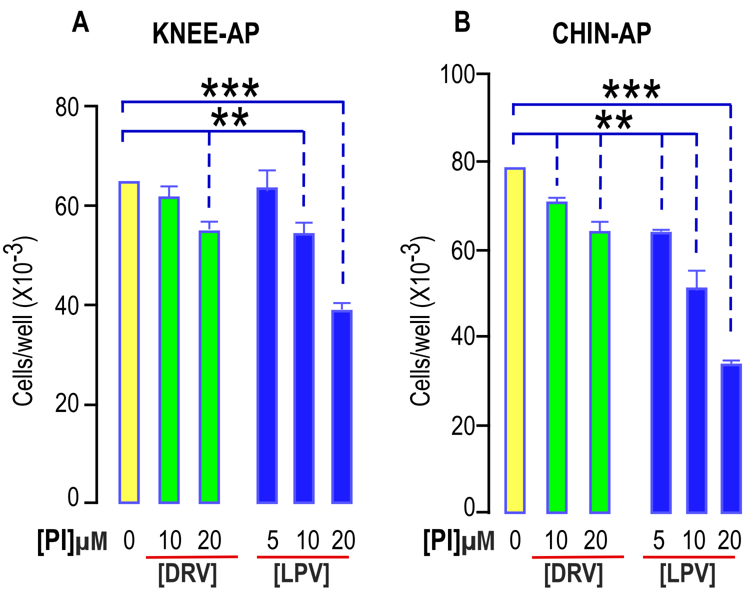

SUPPLEMENTARY FIGURE 3

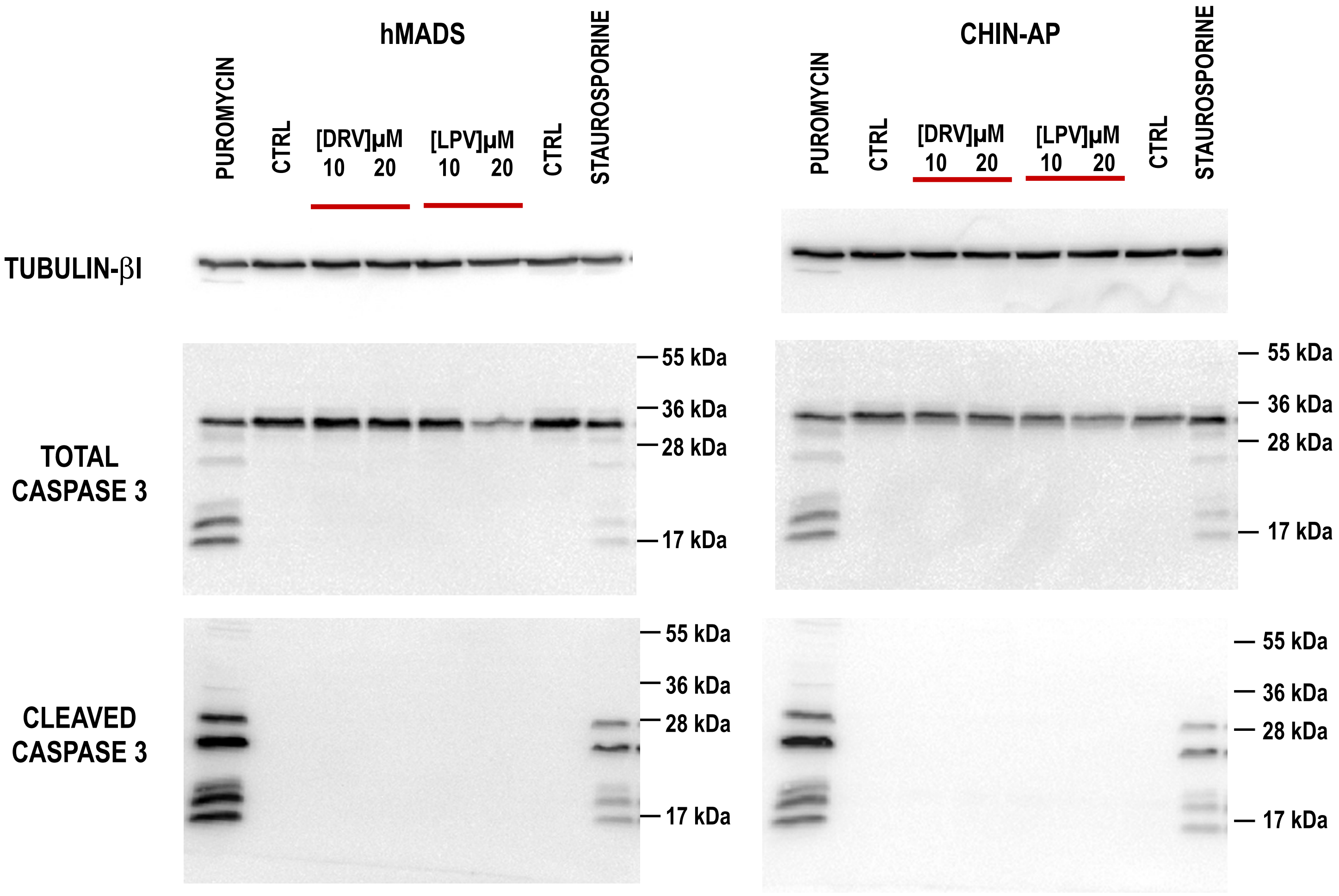

SUPPLEMENTARY FIGURE 4

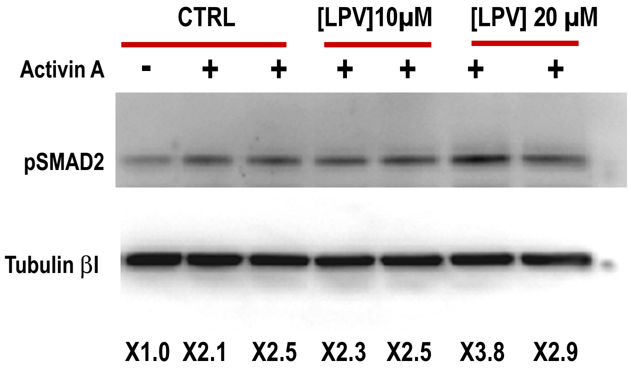

Full length gels for figure 1

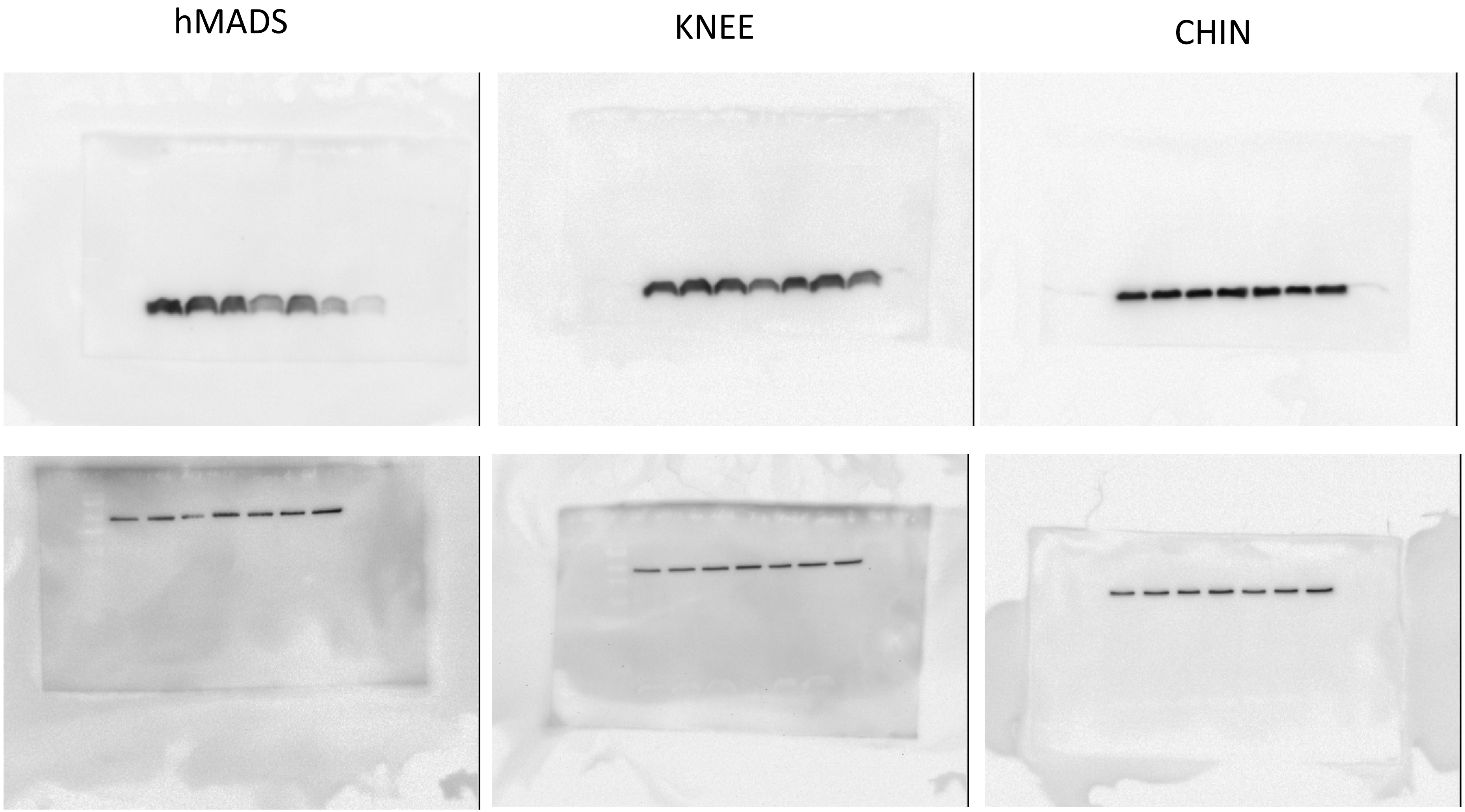

Upper panels : FABP4
Lower panels : Tubulin βI

Full length gels for figure 4

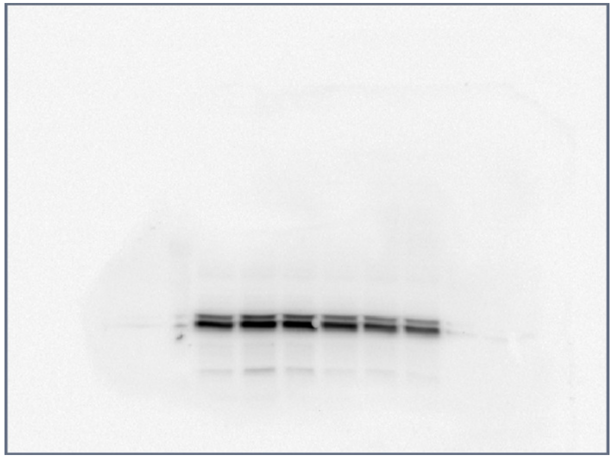

Phospho p42-44

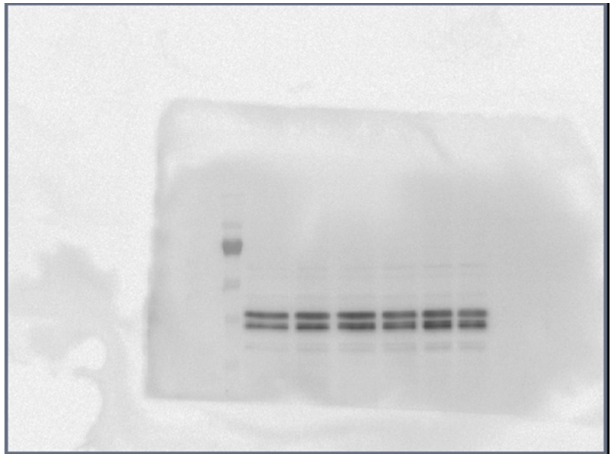

p42-44 TOTAL

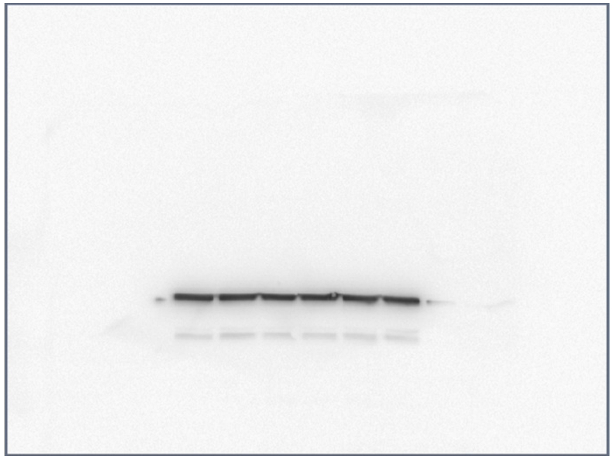

Tubulin-βI

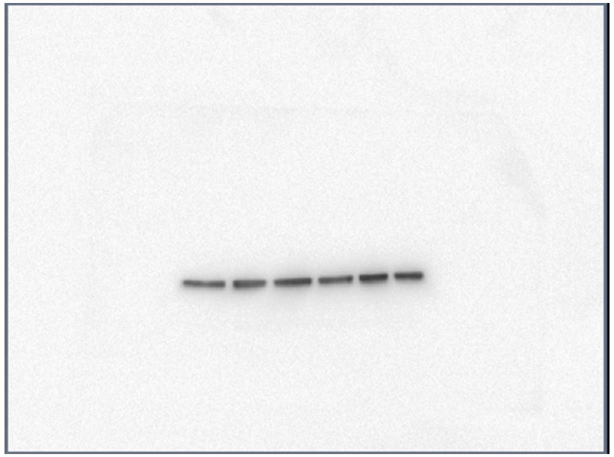

Tubulin-βI

Full length gels for figure 5

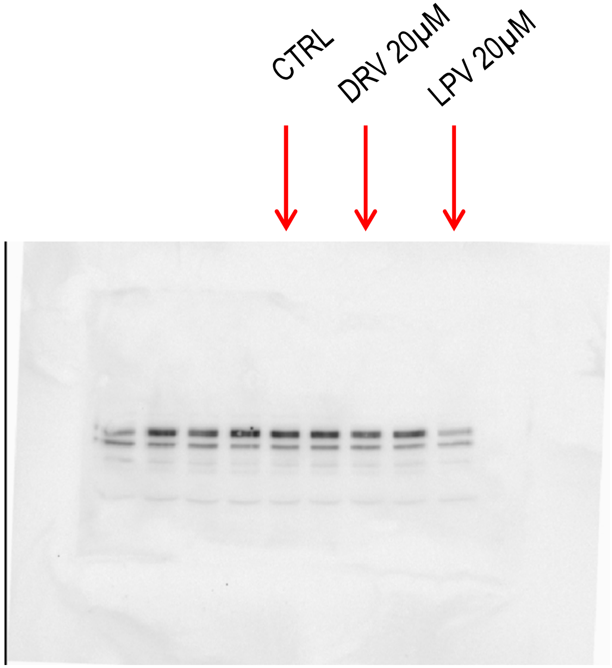

EGR1

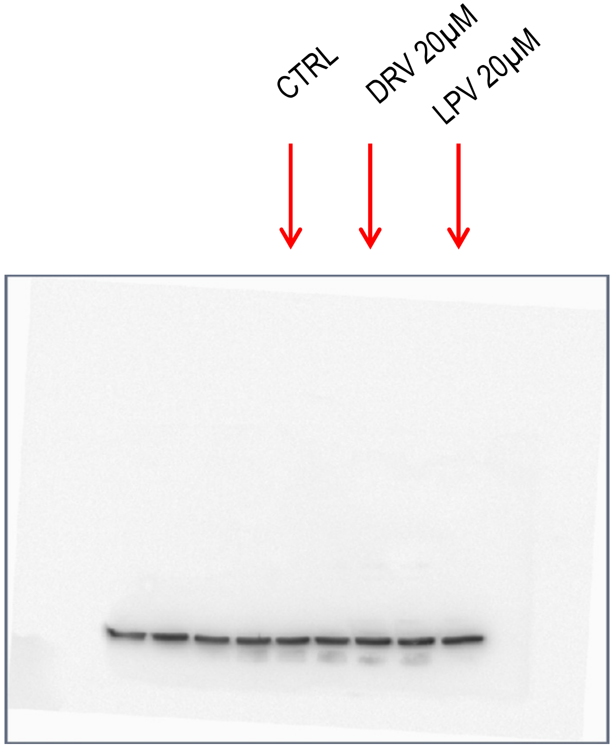

Tubulin-βI

Full length gels for figure 6

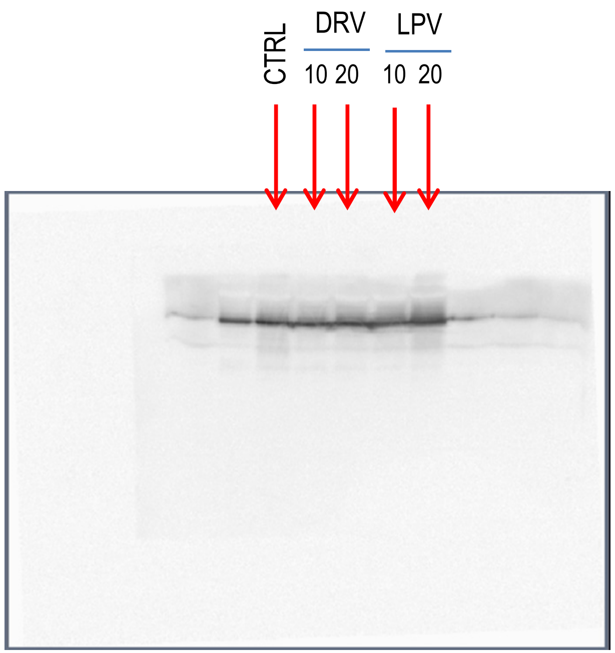

ENOLASE 1

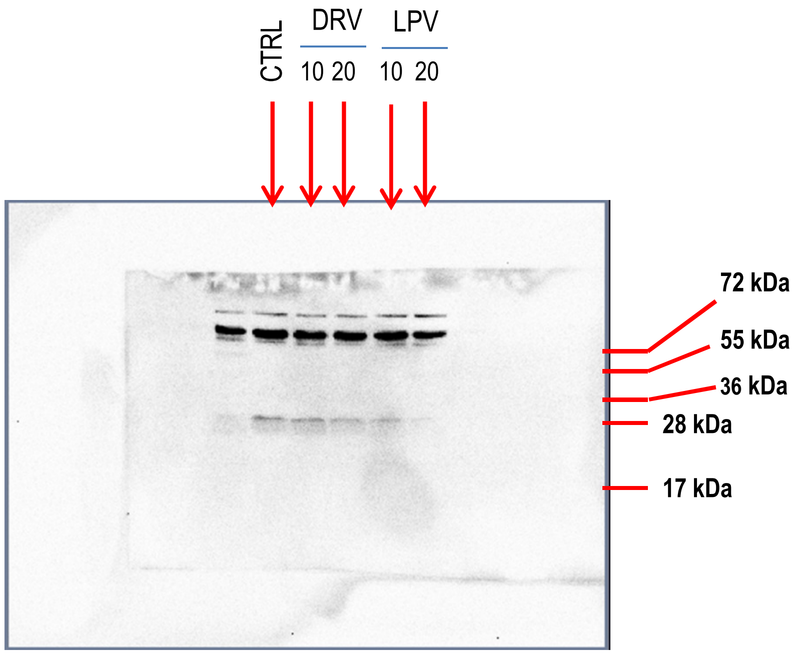

Activin A

Full length gels for supplementary figure 3

hMADS

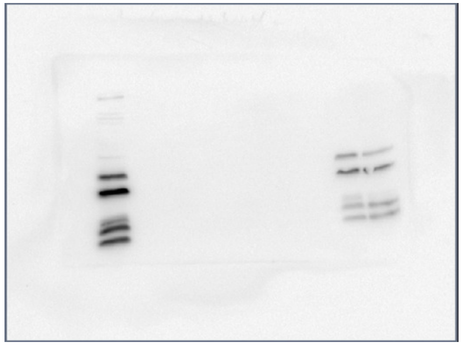

CLEAVED CASPASE 3

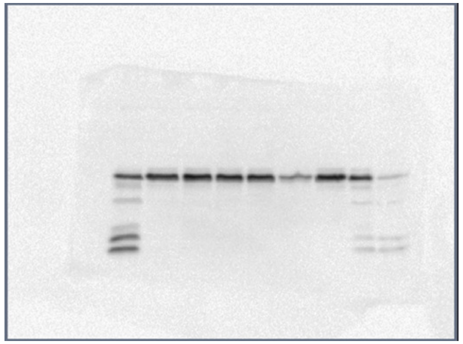

TOTAL CASPASE 3

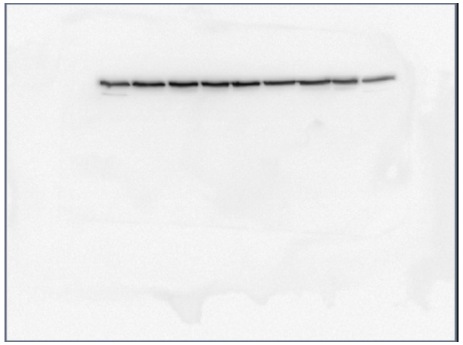

Tubulin-βI

CHIN-AP

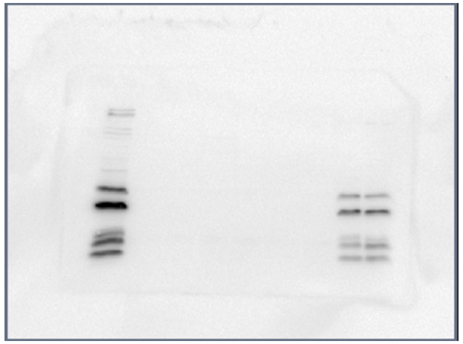

CLEAVED CASPASE 3

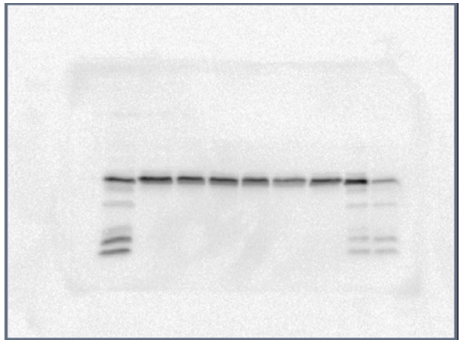

TOTAL CASPASE 3

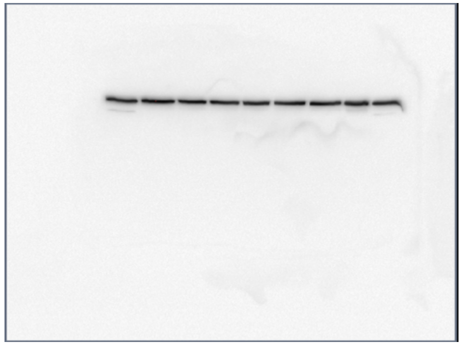

Tubulin-βI

Full length gels for supplementary figure 4

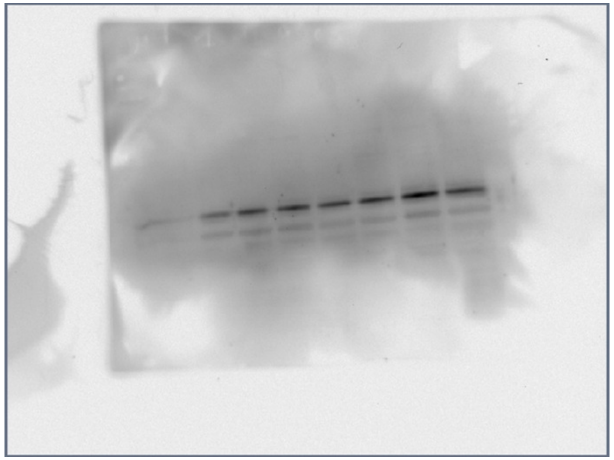

Phospho SMAD 2

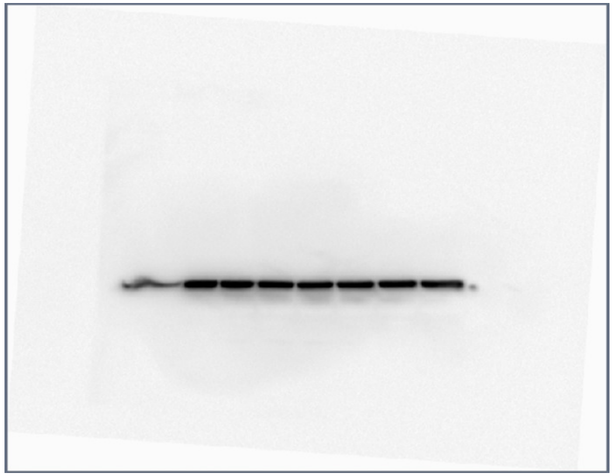

Tubulin-βI
